# Linking signaling pathways to histone acetylation dynamics in plants

**DOI:** 10.1093/jxb/eraa202

**Published:** 2020-04-25

**Authors:** Jianjun Jiang, Adeline B Ding, Fengquan Liu, Xuehua Zhong

**Affiliations:** 1 Institute of Plant Protection, Jiangsu Academy of Agricultural Sciences, Jiangsu Key Laboratory for Food Quality and Safety-State Key Laboratory Cultivation Base of Ministry of Science and Technology, Nanjing, Jiangsu, China; 2 Laboratory of Genetics & Wisconsin Institute for Discovery, University of Wisconsin-Madison, Madison, WI, USA; 3 University Clermont Auvergne, France

**Keywords:** Gene expression, HAT, HDAC, histone acetylation, histone deacetylation, signaling

## Abstract

As sessile organisms, plants face versatile environmental challenges and require proper responses at multiple levels for survival. Epigenetic modification of DNA and histones is a conserved gene-regulatory mechanism and plays critical roles in diverse aspects of biological processes, ranging from genome defense and imprinting to development and physiology. In recent years, emerging studies have revealed the interplay between signaling transduction pathways, epigenetic modifications, and chromatin cascades. Specifically, histone acetylation and deacetylation dictate plant responses to environmental cues by modulating chromatin dynamics to regulate downstream gene expression as signaling outputs. In this review, we summarize current understandings of the link between plant signaling pathways and epigenetic modifications with a focus on histone acetylation and deacetylation.

## Teaser

This review summarizes and highlights the latest advances of histone acetylation dynamics in various systemic and environment-responsive signaling pathways in plants.

## Introduction

Within the nucleus of eukaryotic cells, DNA wraps around histone octamers in a left-handed superhelix to form highly conserved repeating nucleoprotein complexes called nucleosome cores. Each nucleosome core contains a 145–147 bp stretch of DNA and two copies of each core histone, H2A, H2B, H3, and H4 (Luger *et al.*, 1997). A nucleosome core together with its linker DNA and linker histone H1 constitutes a nucleosome. Nucleosomes are the basic subunits of chromatin (Schalch *et al.*, 2005). N-terminal histone tails and DNA are subject to various modifications acting as important mechanisms for gene regulation, genome stability, and genome defense in eukaryotes (Kouzarides, 2007; Zhang *et al.*, 2007; Chinnusamy and Zhu, 2009; Portela and Esteller, 2010). The primary form of DNA modification is methylation at cytosine and adenine residues (Chinnusamy and Zhu, 2009; Portela and Esteller, 2010). Histones are subject to a number of post-translational modifications, including acetylation, methylation, phosphorylation, ubiquitination, and sumoylation (Kouzarides, 2007). While histone acetylation and methylation are among the best characterized modifications, new studies continue to shed light on the mechanisms and functions of other histone modifications.

Accumulating studies have shown the establishment of dynamic epigenetic modifications in response to various environmental stimuli to be an essential mechanism for signal-induced transcription (Suganuma and Workman, 2011; Yamamuro *et al.*, 2016; Ueda and Seki, 2020). For example, bacterial infection by *Pseudomonas syringae* DC3000 (*Pst* DC3000) triggers both DNA hypomethylation and hypermethylation at thousands of genes, among which many are correlated with expression changes in Arabidopsis (Dowen *et al.*, 2012; Yu *et al.*, 2013). Histone modification patterns also change in response to endogenous hormone signaling. The ATP-dependent chromatin remodeler PICKLE is involved in the gibberellin (GA) signaling pathway and regulates GA-mediated developmental processes (Park *et al.*, 2017). Both GA signaling and nitrogen regulate the abundance of NITROGEN-MEDIATED TILLER GROWTH RESPONSE5 (NGR5) which is thought to recruit the POLYCOMB REPRESSIVE COMPLEX2 (PRC2) complex to deposit H3K27me3 in rice (*Oryza sativa*) (Wu *et al.*, 2020). In brassinosteroid (BR) signaling, the transcription factor BRASSINAZOLE-RESISTANT1 (BZR1) is activated in response to BR and recruits the H3K27 demethylase EARLY FLOWERING6 (ELF6). This process removes H3K27me3 at the *FLOWERING LOCUS C* (*FLC*) locus, thus promoting *FLC* expression and inhibiting flowering (Li *et al.*, 2018). Abscisic acid (ABA) signaling and drought stress can induce alterations in histone H2B mono-ubiquitination levels in rice through the interaction between the ABA-activated transcription factor OsbZIP46 and the E3 ubiquitin ligase HISTONE MONO-UBIQUITINATION2 (OsHUB2) (S. Ma *et al.*, 2019). Over the last decade, a large number of studies have demonstrated functional links between histone (de)acetylation and various signaling pathways. In this review, we summarize the current understanding of the interplay between signaling transduction pathways and chromatin dynamics, with a focus on histone acetylation and deacetylation.

## Role of histone deacetylation in plant signaling

Histone acetylation on positively charged lysine residues leads to neutralization of charges. This weakens the interactions between the histone and DNA, thus resulting in open chromatin that is more accessible to regulators (Roth *et al.*, 2001). Additionally, histone acetylation can affect interactions between histones themselves or between histones and regulators (Roth *et al.*, 2001). Removal of acetyl groups by histone deacetylases (HDACs) usually serves as a transcriptional repression mechanism (Shahbazian and Grunstein, 2007). In Arabidopsis, there are a total of 18 HDACs that can be classified into (i) Reduced Potassium Dependence3/Histone Deacetylase-1 (RPD3/HDA1) superfamily, including Class I (HDA6, HDA7, HDA9, HDA10, HDA17, and HDA19), Class II (HDA5, HDA8, HDA14, HDA15, and HDA18), and Class IV (HDA2); (ii) NAD-dependent Sirtuin-like HDACs (Class III, SRT1 and SRT2); and (iii) plant-specific HD2-type HDACs (HD2A, HD2B, HD2C, and HD2D) (Pandey *et al.*, 2002). As HDACs lack the ability to directly bind DNA, they are usually recruited to chromatin by interacting with DNA/chromatin binding partners (Kagale *et al.*, 2010; Liu *et al.*, 2014). In recent years, emerging studies have shown that HDACs play crucial roles in various signaling pathways and physiological processes (Chen *et al.*, 2020; Ueda and Seki, 2020).

### Histone deacetylases in temperature response

The HDACs HDA9, HDA15, and HD2C are reported to be involved in temperature response. Recent studies have shown that HDA9 forms a core repressive complex with the SANT (Swi3, Ada2, N-Cor, and TFIIIB) domain-containing protein POWERDRESS (PWR) and the WD40 repeat protein HIGH EXPRESSION OF OSMOTICALLY RESPONSIVE GENES15 (HOS15) (Chen *et al.*, 2016; Kim *et al.*, 2016; Suzuki *et al.*, 2018; Mayer *et al.*, 2019; Park *et al.*, 2019). This HDA9–PWR–HOS15 complex resembles the HDAC3–NCoR/SMRT–TBL1 transcriptional repression complex in mammals (Karagianni and Wong, 2007). A notable phenotype of *hda9*, *hos15*, and *pwr* mutants is slightly bulged silique tips. Interestingly, the double mutant *hda6 hda9* displays a more severe ‘notch-shaped’ bulge, suggesting that HDA9 and HDA6 share functional redundancy (Kim *et al.*, 2016; Yuan *et al.*, 2019). This is consistent with the finding that HDA9 interacts with HDA6 and HDA19 (Zheng *et al.*, 2016).

**Fig. 1.**
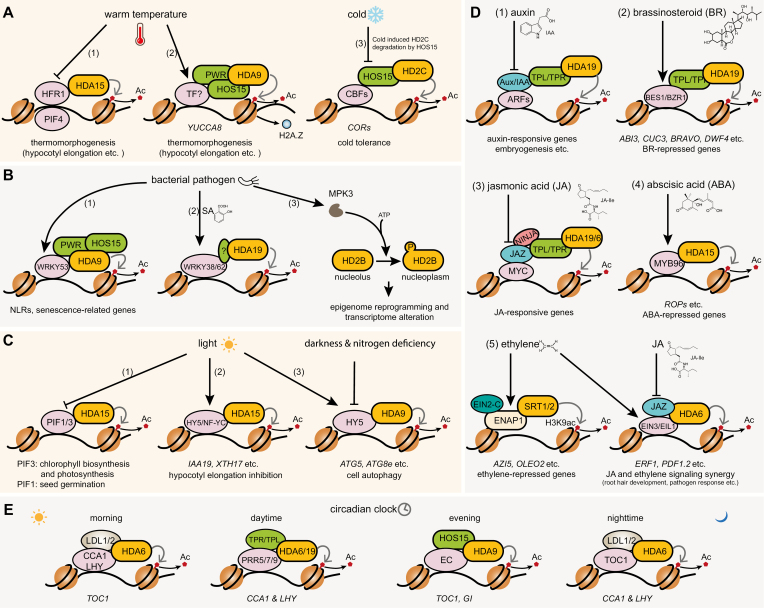
Histone deacetylases in signaling cascades. (A) Histone deacetylases in temperature response. (1) HFR1 associates with HDA15 to repress thermal-responsive genes. Warm temperatures induce PIF4 to compete with HFR1 and inhibit the association of HDA15 at thermal-responsive genes. (2) At warm temperatures, the HDA9–PWR–HOS15 complex induces hypoacetylation at the *YUCCA8* locus and facilitates the eviction of histone variant H2A.Z, resulting in a permissive chromatin environment. (3) At normal temperatures, HOS15, CBFs, and HD2C form a repressive complex targeting *COR* genes. Cold temperatures induce HD2C degradation by the HOS15-mediated proteasome pathway. (B) Histone deacetylases in bacterial pathogen response. (1) Bacterial infection induces the expression of WRKY53, which recruits the HDA9–PWR–HOS15 complex to suppress expression of NLR genes and leaf senescence genes. (2) Bacterial infection induces salicylic acid (SA) production and WRKY38/62 expression. WRKY38/62 recruits HDA19 to fine-tune basal defense responses. (3) Upon recognition of bacterial flagellin at the cell surface, the protein kinase MPK3 phosphorylates HD2B. Phosphorylated HD2B re-localizes from the nucleolus to the nucleoplasm and modulates transcriptome alteration and epigenome reprogramming. (C) Histone deacetylases in light signaling. (1) Under dark conditions, HDA15 associates with PIF3 and PIF1 to mediate H3 and H4 deacetylation and repress the expression of chlorophyll biosynthetic/photosynthetic and seed germination genes. In light conditions, PIFs are phosphorylated and degraded, releasing HDA15 from chromatin and transcriptional repression of these genes. (2) In light, HY5 and NF-YCs interact with HDA15 at the promoters of auxin biosynthetic and signaling genes (e.g. *IAA19*) and cell wall organization-related genes (e.g. *XTH17*) to inhibit expression. (3) Under light, HY5 interacts with HDA9 at *ATG5* and *ATG8e* loci to inhibit cell autophagy. Darkness and nitrogen deficiency induce HY5 degradation to release the inhibition on *ATG5* and *ATG8e*, leading to induction of autophagy. (D) Histone deacetylases in hormone signaling. (1) In the absence of auxin, the repression of auxin-responsive genes by Aux/IAA–TPL/TPR requires deacetylation by HDA19. Auxin induces Aux/IAA protein degradation and thus releases the transcription factors ARFs. (2) BR promotes the nuclear accumulation and DNA binding activity of BES1 and BZR1. BES1/BZR1 recruits the TPL/TPR–HDA19 complex via the EAR motifs to repress the expression of BR-repressed genes. (3) In the absence of JA, JAZ and NINJA interact with TPL/TPR, which further recruits HDA19 and HDA6 for histone deacetylation and transcriptional repression. In the presence of JA, JAZ is degraded, releasing MYC-mediated transcription repression. (4) High levels of ABA promote the interaction between MYB96 and HDA15 to induce H3 and H4 hypoacetylation and regulate ABA-repressed genes (e.g. *ROP* genes). (5) Ethylene promotes the nuclear import of EIN2-C to facilitate its interaction with ENAP1–SRT1/2 to attenuate H3K9ac in ethylene-repressed genes. Ethylene stabilizes the transcription factors EIN3/EIL1, which are repressed by JAZ and HDA6. JA-induced JAZ degradation releases the repression of ethylene-responsive genes. (E) Histone deacetylases in the circadian clock. HDA6 and LDL1/2 interact with CCA1/LHY to repress the expression of *TOC1* in the morning. During the daytime, PPR5/7/9 recruit TPL/TPR and HDA6/19 to repress *CCA1/LHY* expression. In the evening, the Evening Complex (EC) interacts with HDA9 and HOS15 to repress *TOC1* and *GI* expression. At nighttime, HDA6 and LDL1/2 interact with TOC1 to repress the expression of *CCA1/LHY*.

PWR and HDA9 have also been identified as essential components in the ambient temperature-sensing pathway and thermomorphogenesis (Fig. 1A) (Tasset *et al.*, 2018; van der Woude *et al.*, 2019). Both *pwr* and *hda9* mutants show attenuated warm temperature-induced hypocotyl elongation. Treatment with the HDAC inhibitor trichostatin A (TSA) diminishes temperature-induced hypocotyl elongation, suggesting that thermomorphogenesis requires deacetylase activity. At high temperatures, the PWR–HDA9 complex maintains low H3K9/K14 acetylation levels to facilitate the eviction of the histone variant H2A.Z from the nucleosome, thus providing a permissive chromatin environment for the basic helix–loop–helix (bHLH) factor Phytochrome-Interacting Factor4 (PIF4). PIF4 binds to the promoter of *YUCCA8* (a key auxin biosynthesis gene) and activates *YUCCA8* expression and auxin biosynthesis (van der Woude *et al.*, 2019). Since HDA9 does not directly interact with PIF4 (van der Woude *et al.*, 2019), it is unclear how the HDA9 complex is recruited to *YUCCA8.* It is possible that an as yet unidentified high temperature-responsive transcription factor may interact with HDA9 to achieve this function. HDA9–PWR is also involved in high temperature-induced seed germination suppression mediated by the interaction between ABA INSENSITIVE3 (ABI3) and PWR (Yang *et al.*, 2019). However, whether HOS15 also participates in these signaling processes requires further investigation.

Like HDA9, HDA15 is also involved in ambient temperature response with a different role (Shen *et al.*, 2019). HDA15 directly represses thermal-responsive genes at normal temperatures by interacting with the transcription factor LONG HYPOCOTYL IN FAR-RED1 (HFR1), which antagonizes the thermal response regulator PIF4. Elevated temperature reduces the association of HDA15 with warm temperature-responsive genes, probably due to competing with HFR1 by warm temperature-induced PIF4 (Fig. 1A) (Shen *et al.*, 2019).

HD2C is a member of the plant-specific HD2-type HDACs that are involved in growth, development, and response to various stresses (Pandey *et al.*, 2002; Luo *et al.*, 2012; Buszewicz *et al.*, 2016; Han *et al.*, 2016). Under normal temperatures, HD2C interacts with HOS15 and the master cold-responsive transcription factors C-REPEAT BINDING FACTORSs (CBFs) to repress the expression of *COLD-RESPONSIVE* (*COR*) genes. Upon cold treatment, HD2C is ubiquitinated by an E3 ubiquitin ligase complex containing HOS15 and degraded via the proteasome system, leading to *COR* chromatin hyperacetylation (Fig. 1A). Meanwhile, CBFs are rapidly and transiently induced by low temperatures to promote *COR* expression and confer cold tolerance (Park *et al.*, 2018).

### Histone deacetylases in pathogen response

In addition to the temperature-sensing pathway, HDA9 is also involved in other signaling pathways. HDA9 plays important roles in flowering, aging, salt and drought stress response, leaf development, seed germination, and plant immunity (Kang *et al.*, 2015; Chen *et al.*, 2016; Kim *et al.*, 2016; Zheng *et al.*, 2016; Suzuki *et al.*, 2018; Mayer *et al.*, 2019; L. Yang *et al.*, 2020). HDA9 interacts with the transcription factor WRKY53 to bind leaf senescence-associated genes and Nod-Like Receptor (NLR) genes involved in plant immunity (Chen *et al.*, 2016; L. Yang *et al.*, 2020). Bacterial infection with *Pseudomonas syringae* has been shown to induce *WRKY53* expression (Murray *et al.*, 2007; Hu *et al.*, 2012). Thus, it is possible that WRKY53 may recruit the HDA9 complex to genes involved in pathogen-induced leaf senescence and defense responses (Fig. 1B). Recently, WRKY53 was reported to be acetylated. This acetylation can be removed by HDA9, resulting in the inhibition of the DNA binding and transcriptional activity of WRKY53 and the negative regulation of the salt stress response (Zheng *et al.*, 2020).

HDA19 is also involved in plant defense response to pathogens. It is one of the most extensively studied HDACs and acts as a general regulator of chromatin dynamics implicated in various molecular and physiological processes (Long *et al.*, 2006; Liu *et al.*, 2014; Yamamuro *et al.*, 2016; Ning *et al.*, 2019). HDA19 interacts with two transcription factors, WRKY38 and WRKY62, whose expression is induced by salicylic acid (SA), to fine-tune basal defense responses (Kim *et al.*, 2008). High expression and histone acetylation levels of *PATHOGENESIS-RELATED1/2* (*PR1/2*), coupled with increased SA content and resistance to the *Pst* DC3000 infection in *hda19*, suggest that HDA19 acts as a negative regulator of SA-dependent pathogen resistance genes such as *PR1* (Choi *et al.*, 2012). The transcription factor recruiting HDA19 to *PR* gene loci, however, has yet to be identified.

HD2B plays a role in mediating microbial-associated molecular pattern (MAMP)-triggered immunity signaling (Latrasse *et al.*, 2017). Upon perception of flagellin from *Pst* DC3000 at the cell surface, the protein kinase MITOGEN-ACTIVATED PROTEIN KINASE 3 (MPK3) interacts with and phosphorylates HD2B. Phosphorylated HD2B shuttles from the nucleolus to the nucleoplasm (Fig. 1B) (Latrasse *et al.*, 2017). This MPK3–HD2B module controls the basal expression of defense genes. When treated with bacterial flagellin, H3K9 acetylation changes at a genome-wide level due to MPK3–HD2B activity. Consistently, the *hd2b* mutant is more susceptible to *Pst* DC3000 (Latrasse *et al.*, 2017). Interestingly, HD2B and HD2C form a homo- or hetero-oligomer, and are also involved in rRNA processing and biogenesis (Chen *et al.*, 2018).

### Histone deacetylases in light signaling

HDA15 is the main HDAC involved in light signaling pathways through regulating chlorophyll biosynthesis, photosynthesis gene expression, hypocotyl elongation, and seed germination (Liu *et al.*, 2013; Gu *et al.*, 2017; Tang *et al.*, 2017; Ali *et al.*, 2019; Zhao *et al.*, 2019). HDA15 is a Class II HDAC and localizes to both the nucleus and cytoplasm (Alinsug *et al.*, 2012). Interestingly, HDA15 nuclear localization has been shown to be induced by light (Alinsug *et al.*, 2012). Under dark conditions, the transcription factor PIF3 interacts with HDA15 and recruits it to the promoters of chlorophyll biosynthetic and photosynthetic genes, leading to H3/H4 hypoacetylation and transcription repression. In the presence of light, PIF3 is phosphorylated and subsequently degraded, releasing HDA15 from repressing these genes (Liu *et al.*, 2013). Similarly, under dark conditions, HDA15 interacts with PIF1 to repress the expression of genes involved in seed germination by reducing H3 acetylation levels (Gu *et al.*, 2017). The inhibition of hypocotyl elongation is another light-responsive phenotype. Under light, Nuclear Factor-YC homologs (NF-YCs) and ELONGATED HYPOCOTYL5 (HY5) interact with HDA15, targeting it to the promoters of hypocotyl elongation-related genes involved in auxin signaling and cell wall organization to repress their expression (Fig. 1C) (Tang *et al.*, 2017; Zhao *et al.*, 2019). Recently, a new study reported that HY5 also interacts with HDA9 to mediate dark-induced cell autophagy (C. Yang *et al.*, 2020). In light conditions, HY5 recruits HDA9 to autophagy-related genes (*ATG5* and *ATG8e*) to initiate H3K9/K27 deacetylation and transcription suppression. Darkness induces HY5 degradation and dissociates HDA9 from *ATG5* and *ATG8e* chromatin to release the transcriptional inhibition (Fig. 1C). Similarly, nitrogen deficiency induces HY5 degradation and *ATG5* and *ATG8e* expression (C. Yang *et al.*, 2020). However, it should be noted that another study provides evidence that HY5 mainly works as a transcriptional activator during de-etiolation (under light), relying on other factors likely to be regulated by light (Burko *et al.*, 2020). Thus, the precise roles of HY5 and HDACs in light responses need more in-depth investigation.

### Histone deacetylases in plant hormone signaling

HDA19 is involved in multiple plant hormone signaling pathways (Long *et al.*, 2006; Liu *et al.*, 2014; Yamamuro *et al.*, 2016; Ning *et al.*, 2019). HDA19 functions in complex with the plant Groucho/Tup1 co-repressor TOPLESS/TOPLESS-RELATED (TPL/TPR), which consists of an N-terminal LisH (lissencephaly homology) domain and C-terminal WD40 repeats (Zhu *et al.*, 2010; Krogan *et al.*, 2012). The *hda19* mutants exhibit strong *tpl-1* mutant-like phenotypes at 29 °C, including monocots, tubes, and pins, suggesting that TPL and HDA19 act in the same genetic pathway (Long *et al.*, 2006). However, a direct TPL–HDA19 interaction has not been observed, indicating a requirement for an adaptor protein (Causier *et al.*, 2012).

TPL/TPR co-repressors function through interactions with Ethylene response factor-associated Amphiphilic Repression (EAR) motifs that are found in many transcription factors (Causier *et al.*, 2012). EAR motifs have sequence patterns of LXLXL or DLNXXP (X represents any amino acid). Artificial fusion of an EAR motif (LDLDLELRLGFA, SRDX) with transcription factors is sufficient to suppress target gene expression, making this an ideal tool for overcoming genetic redundancy (Hiratsu *et al.*, 2003). Many transcription factors or cofactors containing EAR motifs interact with the TPR/TPL–HDA19 complex to mediate transcriptional responses to upstream signaling (Kagale and Rozwadowski, 2011; Yang *et al.*, 2018). In auxin signaling, AUXIN RESPONSE FACTOR (ARF) transcription factors bind DNA to regulate transcription of auxin-responsive genes and are repressed by the AUXIN/INDOLE-3-ACETIC ACID (Aux/IAA) repressors. In the absence of auxin, the Aux/IAA protein IAA12/BODENLOS (IAA12/BDL) interacts with TPL, which associates with HDA19 to repress auxin-responsive genes important for embryogenesis, especially at higher temperatures (e.g. 29 °C) (Fig. 1D) (Long *et al.*, 2006; Szemenyei *et al.*, 2008). Additional Aux/IAA proteins (IAA1–20, 26–32, 34) and repressive ARFs (ARF2 and ARF9) that contain EAR motifs and interact with TPL/TPRs have been identified, and the crystal structures of TPR2 in complex with the EAR motifs from IAA1 and IAA10 have been solved (Kagale *et al.*, 2010; Causier *et al.*, 2012; Ke *et al.*, 2015). Acting as ‘molecular glue’, auxin promotes the interactions between co-receptors Aux/IAA and the TRANSPORT INHIBITOR RESPONSE1/AUXIN SIGNALING F-BOX PROTEIN (TIR1/AFB) family of F-box proteins, leading to the ubiquitination and degradation of Aux/IAA proteins and release of repression of ARFs by TPL/TPR and HDA19 (Lavy and Estelle, 2016). In the BR signaling pathway, the master transcription factors, BRI1-EMS-SUPPRESSOR1 (BES1) and BZR1, also contain EAR motifs that are critical for their interactions with TPL/TPRs and HDA19 in a BR-enhanced manner (Oh *et al.*, 2014; Ryu *et al.*, 2014). In both the *bes1-D* gain-of-function mutant and BR-treated plants, the H3 acetylation level on the *ABI3* promoter is decreased, accompanied by decreased *ABI3* expression, reduced ABA sensitivity, and early flowering (Ryu *et al.*, 2014; Hong *et al.*, 2019). A triple *tpl* mutant (*tpl;tpr1;tpr4*) showed reduced BR sensitivity and suppressed the gain-of-function *bzr1-1D* mutant phenotype. Consistently, TSA treatment decreases sensitivity to BR, suggesting an essential role for HDAC in BR response (Oh *et al.*, 2014). TPL also targets *CUP SHAPED COTYLEDON3* (*CUC3*), *BRASSINOSTEROIDS AT VASCULAR AND ORGANIZING CENTER* (*BRAVO*), and *DWARF4* (*DWF4*) via BES1/BZR1 to regulate organ boundary formation, root quiescent center cell division, and the BR feedback regulation, respectively (Fig. 1D) (Espinosa-Ruiz *et al.*, 2017; Kim *et al.*, 2019).

In addition to auxin and BR, the transcriptional regulators of numerous other plant hormones have been reported to contain EAR motifs and interact with TPL/TPRs (Fig. 1D), including JASMONATE ZIM DOMAIN (JAZ) and NOVEL INTERACTOR OF JAZ (NINJA) in jasmonic acid (JA) signaling (Pauwels *et al.*, 2010; Causier *et al.*, 2012; Shyu *et al.*, 2012), DWARF53/SUPPRESSOR OF MORE AXILLARY GROWTH2-LIKE (D53/SMXLs) in strigolactone signaling (Wang *et al.*, 2015; Liang *et al.*, 2016), ABI5 BINDING PROTEIN (AFPs) in ABA signaling, and NIM1-INTERACTING1/3 (NIMIN1/3) in SA signaling (Kagale *et al.*, 2010; Causier *et al.*, 2012; Ke *et al.*, 2015). However, whether HDA19 or other HDACs function together with these transcriptional regulator–TPL/TPRs complexes requires further investigation.

The TPL/TPR co-repressors and HDA19 are also involved in developmental processes. For example, the transcription factor WUSCHEL HOMEOBOX5 (WOX5) functions with TPR/TPL and HDA19 to silence the differentiation factor *CYCLING DOF FACTOR4* (*CDF4*) to maintain the undifferentiated status of root stem cells (Pi *et al.*, 2015). The transcriptional factor WUSCHEL (WUS) rheostatically controls the auxin signaling by regulating histone acetylation (H3K9/K14) and expression of genes in the auxin signaling pathway to maintain shoot stem cells (Y. Ma *et al.*, 2019).

HDA6, a close homolog to HDA19, is also involved in hormone signaling. HDA6 interacts with both ethylene-stabilized master transcription factors, ETHYLENE INSENSITIVE 3 (EIN3) and EIN3-LIKE 1 (EIL1), and JA-degraded JAZ to regulate JA-induced derepression of ethylene-responsive genes (Zhu *et al.*, 2011). Thus, HDA6 connects the crosstalk between ethylene and JA (Fig. 1D). As HDA6 shares sequence similarity with HDA19 and HDA9, and also interacts with TPL/TPRs (Wang *et al.*, 2013), it is possible that HDA6 may act similarly to HDA19 in these signaling processes. Besides signaling, HDA6 has been shown to play important roles in DNA methylation, gene silencing, nuclear dominance, leaf development, flowering, circadian clock rhythms (as discussed hereafter), and other biological processes (Chen *et al.*, 2020).

Several other HDACs such as HDA15 and SRTs are also involved in plant hormone signaling. HDA15 participates in ABA signaling by interacting with the transcription factor MYB96. When the ABA level is high, MYB96 promotes transcription of ABA-induced genes, but requires HDA15 to repress the transcription of ABA-repressed *RHO OF PLANTS* (*ROP*) genes through promoting H3 and H4 deacetylation at their promoters (Fig. 1D) (Lee and Seo, 2019). HDA9 is also involved in ABA signaling and drought response. The *hda9* mutant is insensitive to ABA but sensitive to drought stress. During drought stress, the transcription factor ABI4 interacts with HDA9 to repress *CYP707A1* and *CYP707A2*, which encode key enzymes catabolizing ABA (Baek *et al.*, 2020). Two NAD^+^-dependent HDACs, SRT1 and SRT2, were recently implicated in the ethylene signaling pathway (Zhang *et al.*, 2018). SRT1 localizes in the nucleus, while SRT2 localizes to either the nucleus or mitochondria depending on different splicing variants (Wang *et al.*, 2010; König *et al.*, 2014; Liu *et al.*, 2017). Though the majority of SRT2 proteins are localized in the mitochondria (König *et al.*, 2014), the double mutant *srt1 srt2* suppresses the constitutive ethylene response phenotypes of the gain-of-function mutants *ENAP1ox* and *EIN3ox*, suggesting that SRT1 and nuclear SRT2 are required for the negative regulation of certain ethylene-responsive genes (Zhang *et al.*, 2018). EIN2 NUCLEAR ASSOCIATED PROTEIN1 (ENAP1) interacts with both SRT1 and SRT2 to reduce H3K9ac levels and suppress transcription at ethylene-repressed genes (Zhang *et al.*, 2018) (Fig. 1D).

### Histone deacetylases in the circadian clock

HDA9 and HDA6 are reported to regulate the circadian clock (Fig. 1E). HDA9 and the Evening Complex (EC) component EARLY FLOWERING3 (ELF3) directly interact to regulate the declining phase of *TIMING OF CAB EXPRESSION 1* (*TOC1*) after its peak expression during the evening. HDA9 specifically binds to the *TOC1* promoter through the interaction with ELF3 to deacetylate H3 at the *TOC1* locus and inhibit *TOC1* transcription at night (Lee *et al.*, 2019). Similarly, HOS15 and HDA9 associate with the EC components LUX ARRHYTHMO (LUX), ELF3, and ELF4 at the *GIGANTEA* (*GI*) promoter to regulate the photoperiodic flowering pathway (Park *et al.*, 2019). During the daytime, PSEUDORESPONSE REGULATOR proteins (PRR5/7/9) interact with TPL/TPR proteins and HDA6/19 at the promoters of the core clock genes *CIRCADIAN CLOCK-ASSOCIATED1* (*CCA1*) and *LATE ELONGATED HYPOCOTYL* (*LHY*) to restrict their expression to near dawn (Wang *et al.*, 2013). The LYSINE-SPECIFIC DEMETHYLASE1 (LSD1)-like histone demethylases, LDL1 and LDL2, interact with HDA6 and CCA1/LHY to repress the expression of *TOC1* in the morning (Hung *et al.*, 2018). In turn, HDA6, LDL1, and LDL2 can also interact with TOC1 to repress *CCA1*/*LHY* expression in the evening (Hung *et al.*, 2019).

## Role of histone acetylation in plant signaling

Opposite to deacetylation, histone acetylation loosens chromatin conformation and generally activates transcription (Shahbazian and Grunstein, 2007). Histone acetylation is catalyzed by histone acetyltransferases (HATs). Eukaryotic HATs can be broadly organized into two classes: nucleus-localized HAT-A and cytoplasm-localized HAT-B (Brownell and Allis, 1996; Roth *et al.*, 2001; Boycheva *et al.*, 2014). HAT-A can be further divided into four types: GNAT (GCN5-related N-terminal acetyltransferases), MYST (MOZ, Ybf2/Sas3, Sas2, and Tip60-related), p300/CREB-binding protein (CBP), and TAF1 (TATA-binding protein-associated factor) (Servet *et al.*, 2010; Boycheva *et al.*, 2014). In Arabidopsis, there are three GNAT family members, including General Control Nondepressible 5 (GCN5, also named HAG1), Elongator complex protein 3 (ELP3, also named HAG3), and HAG2; two MYST family members (HAG4/HAM1 and HAG5/HAM2); five p300/CBP family members (HAC1, HAC2, HAC4, HAC5, and HAC12); and two TAF1 family members (HAF1 and HAF2/TAF1) (Servet *et al.*, 2010; Boycheva *et al.*, 2014). In maize, HAT-B has acetylation activity on free histones, especially H4 (Lusser *et al.*, 1999). In recent years, emerging evidence has demonstrated the important roles of histone acetylation in both upstream signaling transduction pathways and downstream gene expression (Yamamuro *et al.*, 2016; Ueda and Seki, 2020).

### GCN5 in signaling cascades

**Fig. 2.**
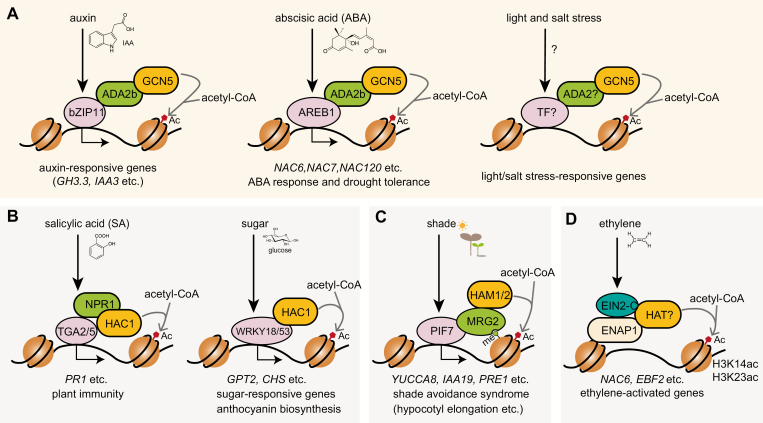
Histone acetyltransferases in signaling cascades. (A) GCN5-mediated histone acetylation. In the presence of auxin, bZIP11 functions as a quantitative modulator to boost auxin-mediated transcription by recruiting the ADA2b–GCN5 complex (left panel). ABA signaling phosphorylates AREB1 and induces its expression. AREB1 then recruits the ADA2b–GCN5 complex to *NAC* genes to induce hyperacetylation and expression (middle panel). In response to light and salt stress, GCN5 interacts with unknown transcription factor(s), probably via an ADA2 adaptor to regulate the expression of light/salt-responsive genes (right panel). (B) HAC1-mediated histone acetylation. Pathogen-induced SA accumulation promotes the formation of the HAC1–NPR1–TGA2/5 complex to induce histone hyperacetylation and transcriptional activation of immunity-responsive genes (e.g. *PR1*, left panel). An increased sugar (glucose) level promotes the association of HAC1 with WRKY18 and WRKY53 to induce H3K27 hyperacetylation and transcription activation of sugar-responsive genes and genes involved in anthocyanin biosynthesis (right panel). (C) HAM in shade response. Under shade, PIF7 is in an active form and interacts with the HAM1/HAM2-associated histone methylation reader MRG2 to induce histone hyperacetylation and expression of genes involved in auxin biosynthesis. (D) HAT in ethylene response. Ethylene promotes the nuclear import of EIN2-C to interact with ENAP1 and an unknown HAT to induce H3K14 and H3K23 hyperacetylation. Acetyl-CoA serves as an acetyl group donor.

GCN5 acetyltransferase forms a complex with the adaptor proteins ADA2a and ADA2b (also named Proporz1/PRZ1) (Mao *et al.*, 2006). The ADA2–GCN5 complex plays important roles in growth, development, and response to stresses including heat, salt, and phosphate starvation (Vlachonasios *et al.*, 2003; Servet *et al.*, 2010; Hu *et al.*, 2015; Wang et al., 2019*a*, *b*; Zheng *et al.*, 2019). As the most extensively studied HAT in plants, the GCN5 complex has been shown to mediate several signaling-induced gene expression pathways. Treatment with the GCN5 inhibitor butyrolactone reduces plant sensitivity to auxin (Weiste and Dröge-Laser, 2014). ADA2b is required for histone acetylation at several auxin-responsive loci, and the *ada2b* mutant is impaired in the morphogenic response to auxin (Anzola *et al.*, 2010). The transcription factor bZIP11 interacts with ADA2b through its N-terminal domain and recruits the ADA2b–GCN5 complex to auxin-responsive genes such as *GH3.3* and *IAA3*, leading to an enhanced H3K27ac level and RNA Pol II recruitment (Weiste and Dröge-Laser, 2014). In ABA signaling, the transcription factor ABA-RESPONSIVE ELEMENT BINDING1 (AREB1, also named ABF2) is phosphorylated by the upstream kinases SNF1-RELATED PROTEIN KINASES 2 (SnRK2s). AREB1 then activates the expression of many ABA-responsive genes by binding to the ABA-Responsive Element (ABRE) of target genes and confers plant drought tolerance (Li *et al.*, 2019). In poplar trees (*Populus trichocarpa*), PtrAREB1 interacts with and recruits the ADA2b–GCN5 complex to drought-responsive genes (e.g. *PtrNAC6*, *PtrNAC7*, and *PtrNAC120*), resulting in enhanced H3K9ac and RNA Pol II enrichment and activated transcription (Fig. 2A) (Li *et al.*, 2019). Consistently, poplar trees with overexpression and knockdown of *AREB1*, *ADA2b*, and *GCN5* are more tolerant and susceptible to drought stress, respectively (Li *et al.*, 2019). The ADA2–GCN5 complex also regulates several developmental processes. For example, the transcription factor WOX11 interacts with the ADA2–GCN5 complex to activate gene expression and regulate crown root meristem development in rice (Zhou *et al.*, 2017).

GCN5 is also involved in other signaling processes, though precise mechanisms are unknown. First, GCN5 is connected to light signaling (Fig. 2A). The *gcn5* mutant displays a long hypocotyl phenotype and reduced light-inducible gene expression. The phenotypes are further enhanced by the mutation of another acetyltransferase, TAF1 (Benhamed *et al.*, 2006). While both GCN5 and TAF1 are required for H3K9, H3K27, and H4K12 acetylation on the target promoters, H3K14 acetylation solely depends on GCN5 (Benhamed *et al.*, 2006). Genome-wide analysis of GCN5 target genes reveals an enrichment in early light-responsive genes (Fig. 2A) (Benhamed *et al.*, 2008). Secondly, under salt stress, Arabidopsis GCN5 targets *CHITINASE-LIKE1* (*CTL1*), *POLYGALACTURONASE INVOLVED IN EXPANSION3* (*PGX3*), and *MYB54* to mediate histone acetylation and transcriptional activation (Fig. 2A). The *gcn5* mutant displays severe growth inhibition and cell wall integrity defects under salt stress conditions (Zheng *et al.*, 2019). Thirdly, GCN5 is required for heat stress response, as the *gcn5* mutant shows impaired induction of heat-responsive genes (Hu *et al.*, 2015). Fourthly, GCN5 is required for pluripotency acquisition (Kim *et al.*, 2018). Epigenetic reprogramming via GCN5 establishes competency for shoot regeneration from a callus by promoting the expression of root stem cell factors. In calli, GCN5 promotes transcription of *WOX*, *SCR*, and *PLT* root stem cell regulators through histone acetylation at their promoters (Kim *et al.*, 2018). Finally, histone acetylation orchestrates wound-induced transcriptional activation and cellular reprogramming (Rymen *et al.*, 2019). Inhibition of GNAT–MYST-mediated histone acetylation by *gcn5* mutation or inhibitor treatment strongly blocks wound-induced transcriptional activation as well as callus formation at wound sites (Rymen *et al.*, 2019).

### HAC1 in signaling cascades

HAC1 is a p300/CBP-type HAT and regulates several signaling processes, as revealed by the pleiotropic phenotypes of *hac1* mutants (Han *et al.*, 2007). In the SA signaling pathway, HAC1 and HAC5 form a complex with the transcription factors TGACG SEQUENCE-BINDING PROTEIN2/5 (TGA2/5) and the master immune regulator NONEXPRESSOR OF PR GENES1 (NPR1). The HAC–NPR1–TGA complex activates SA-dependent plant immunity by promoting *PR* transcription through histone acetylation (Fig. 2B). The double mutant *hac1 hac5* is more susceptible to *Pst* DC3000 infection (Jin *et al.*, 2018). Moreover, pre-treatment with repetitive (≥7 times) abiotic stress (heat, cold, or salt) induces resistance to the virulent bacteria *Pst* DC3000. HAC1 has been implicated in the priming of pattern-triggered immunity (PTI)-induced genes and basal resistance, as the repetitive stress-induced priming is lost in the *hac1* mutant (Tsai *et al.*, 2014). In the sugar signaling pathway, WRKY18 and WRKY53 interact with HAC1 at the W-box element of sugar-induced genes, including GLUCOSE-6-PHOSPHATE/PHOSPHATE TRANSLOCATOR (*GPT2*), DIHYDROFLAVONOL 4-REDUCTASE (*DFR*), and CHALCONE SYNTHASE (*CHS*), resulting in H3K27 hyperacetylation and transcriptional activation (Fig. 2B) (Chen *et al.*, 2019). Both *wrky18 wrky53* and *hac1* mutants showed impaired sugar response as indicated by attenuated anthocyanin accumulation and sugar-responsive gene expression (Chen *et al.*, 2019).

### Other HATs

HAM1 and HAM2 are two MYST-type HATs that catalyze H4K5 acetylation. They have been demonstrated to play important roles in development, as the *ham1/ham2* double mutant is lethal (Latrasse *et al.*, 2008). HAM1/ HAM2 is reported to link shade–light signaling to chromatin acetylation (Fig. 2C) (Peng *et al.*, 2018). Under shade conditions, the transcription factor PIF7 interacts with MORF RELATED GENE2 (MRG2), a histone methylation reader that physically interacts with HAM1/HAM2, to elevate the levels of H4K5ac, H3K9ac, and H3K27ac at the promoters of downstream target genes, including *YUCCA8*, *IAA19*, and PACLOBUTRAZOL RESISTANCE1 (*PRE1*) (Peng *et al.*, 2018). Whether HAM1 and HAM2 directly mediate H4K5ac in this process is unclear.

HAM1 and HAM2 are also required for DNA repair after damage by UV-B (Campi *et al.*, 2012). Arabidopsis mutants *ham1* and *ham2* showed increased DNA damage and cyclobutane pyrimidine dimer (CPD) accumulation after UV-B exposure, suggesting that HAM1 and HAM2 are required for DNA repair (Campi *et al.*, 2012). ELP3/HAG3, another GNAT family protein, is involved in UV-B light response. UV-B treatment was reported to increase H3 acetylation (Qüesta *et al.*, 2010). *HAG3-RNAi* plants displayed low leaf and root growth inhibition by UV-B irradiation, high levels of UV-B-absorbing compounds, and little UV-B-induced DNA damage (Fina and Casati, 2015). Similarly, the TAF1-type acetyltransferase HAF1 may also play a role in UV-B responses, as *haf1* mutants show less growth inhibition by UV-B than the wild type (Fina *et al.*, 2017). Another TAF1-type HAT, HAF2, is involved in both red/far-red and blue light signaling pathways, as *haf2* mutants show decreased chlorophyll accumulation and light-induced gene expression (Bertrand *et al.*, 2005).

Ethylene has been reported to trigger the elevation of histone acetylation at H3K14, H3K23, and H3K9, especially at ethylene-responsive genes (Zhang *et al.*, 2016; Wang *et al.*, 2017). In the presence of ethylene, the C-terminus of ETHYLENE-INSENSITIVE2 (EIN2) is cleaved from the endoplasmic reticulum and transported to the nucleus, where EIN2-C interacts with ENAP1 and triggers histone acetylation (Zhang *et al*., 2016, 2017). The increase of histone acetylation suggests that certain HAT(s) may be recruited to this EIN2-C–ENAP1 complex (Fig. 2D). The *gcn5* mutant shows hypersensitivity to ethylene treatment and elevated H3K9ac and H3K14ac levels in the promoter regions of ethylene response genes that are accompanied by increased gene expression (Poulios and Vlachonasios, 2016). The double mutant *hac1 hac5* also shows hypersensitivity to ethylene constitutive triple response with elevated levels of transcription of ethylene-responsive genes (Li *et al.*, 2014). These findings suggest that HAC1/HAC5 and GCN5 may have indirect roles in ethylene signaling.

Histone acetylation has also been implicated in cold response. The cold-responsive transcription factor CBF1 interacts with ADA2b, and overexpression of CBF1 results in H3 hyperacetylation at *COR* genes (Mao *et al.*, 2006; Pavangadkar *et al.*, 2010). However, *ada2b* and *gcn5* mutants show similar H3 acetylation increases to wild-type plants after cold acclimation (Pavangadkar *et al.*, 2010). The underlying mechanism of histone acetylation in cold response needs to be further investigated.

## Conclusions and perspectives

Through intensive and fruitful studies over the past several decades, it has become clear that epigenetic modifications and chromatin dynamics are integral parts of signaling pathways that bridge the gap between transcription factors, downstream gene regulation, and transcription machinery for signaling output. This review summarizes the recent studies articulating the roles of HDACs and HATs in various signaling pathways (for a list of histone deacetylases and acetyltransferases in this review, see Table 1). Despite the progress in this field, many questions remain unanswered. Compared with the large number of studies describing altered epigenetic modifications in response to signaling pathways, how these modifications are generated, maintained, and transduced remains largely unknown. Knowledge based on genetic and genomic studies provides correlative information, but does not address the underlying mechanisms, as one of the main bottlenecks in the study of the plant HDAC or HAT complexes has been their purification at the biochemical level. Given the large number of HDACs/HATs and the various internal and external signaling pathways, little is known about the extent to which each HDAC/HAT participates in signaling cascades and through what specific mechanisms they function. For example, does HDA9–PWR–HOS15 assemble in a core complex involved within a particular pathway or in all signaling pathways? Do PWR and HOS15 act together with other HDACs (e.g. HDA6) to mediate signaling responses? Besides interacting with specific transcription factors of various signaling pathways, are HATs/HDACs themselves regulated by certain signals for proper subcellular localization, protein stability, catalytic activity, and transcription? Currently, while the majority of studies performed have focused on the steady-state levels of epigenetic modifications, the stimuli-induced chromatin dynamic patterns have largely been uninvestigated. Although altered epigenetic modification patterns in abnormal developmental tissues and in plants exposed to diverse environmental stresses have been well documented, whether these changes are causal or consequential is poorly understood. More work is needed to decipher the nature of the signals that trigger specific epigenetic modifications, the precise mechanisms of how epigenetic modulation of chromatin dynamics and gene expression dictate responses to versatile signaling pathways, and the routes through which environmental conditions feed back to epigenome landscapes.

**Table 1.** List of histone deacetylases and acetyltransferases in this review

Signals/Stresses	Type	Component name	Associated proteins
Warm temperature	Deacetylase	HDA9	PWR, HOS15
	Deacetylase	HDA15	HFR1
Cold	Deacetylase	HD2C	HOS15, CBFs
Bacterial pathogen	Deacetylase	HDA9	WRKY53, HOS15, PWR
	Deacetylase	HDA19	WRKY38/62
	Deacetylase	HD2B	MPK3
Light	Deacetylase	HDA15	PIF1, PIF3, NF-YCs, HY5
	Deacetylase	HDA9	HY5
	Acetyltransferase	GCN5	ADA2a
	Acetyltransferase	TAF1/HAF2	NA
	Acetyltransferase	HAM1/HAM2	PIF7, MRG2
Auxin	Deacetylase	HDA19	TPL/TPR, Aux/IAAs
	Acetyltransferase	GCN5	ADA2b, bZIP11
Brassinosteroid	Deacetylase	HDA19	TPL/TPR, BES1/BZR1
Abscisic acid	Deacetylase	HDA15	MYB96
	Deacetylase	HDA19?	TPL/TPR and AFP
	Deacetylase	HDA9	ABI4
	Acetyltransferase	GCN5	ADA2b, AREB1
Ethylene	Deacetylase	SRT1/2	ENAP1, EIN2
	Acetyltransferase	NA	ENAP1, EIN2
Jasmonic acid	Deacetylase	HDA6/19	TPL/TPR, JAZ, NINJA
Strigolactone	Deacetylase	NA	TPL/TPR, D53/SMXLs
Salicylic acid	Deacetylase	HDA19	WRKY38/WRKY62, TPL/TPR, NIMIN1/3
	Acetyltransferase	HAC1	TGA2/5, NPR1
Circadian clock	Deacetylase	HDA6	TOC1, CCA1/LHY, LDL1/2, PRRs
	Deacetylase	HDA9	HOS15, EC (ELF3)
Sugar	Acetyltransferase	HAC1	WRKY18/53
Salt stress	Acetyltransferase	GCN5	NA
UVB stress	Acetyltransferase	ELP3/HAG3	NA
	Acetyltransferase	HAM1/HAM2	NA
Heat stress	Acetyltransferase	GCN5	NA
Wound	Acetyltransferase	GCN5	NA
